# Evaluation of asymptomatic bacteruria management before and after antimicrobial stewardship program implementation: retrospective study

**DOI:** 10.1186/s12879-021-06460-6

**Published:** 2021-08-25

**Authors:** Ahlam Alghamdi, Majid Almajid, Raneem Alalawi, Amjad Alganame, Shorooq Alanazi, Ghaida Alghamdi, Salman Alharthi, Isra Alghamdi

**Affiliations:** 1 Pharmacy Practice Department, Princess Nourah Bint Abdulrahman University, Riyadh, Saudi Arabia; 2 Pharmacy Practice Department, King Abdullah bin AbdulAziz University Hospital, Riyadh, Saudi Arabia; 3 Department of Pharmacy, Security Forces Hospital Program, Riyadh, Saudi Arabia; 4 College of Pharmacy, Princess Nourah Bint Abdulrahman University, Riyadh, Saudi Arabia; 5 College of Medicine, Imam Mohammed Ibn Saud Islamic University, Riyadh, Saudi Arabia

**Keywords:** Antimicrobial agent, Asymptomatic bacteriuria, Urinary tract infection, Antimicrobial stewardship program

## Abstract

**Background:**

The Infectious Diseases Society of America (IDSA) recommends against screening for and/or treating asymptomatic bacteriuria (ASB). This study aims to evaluate the inappropriate use of antibiotics in ASB before and after Antimicrobial Stewardship Program (ASP) implementation and advance towards its appropriate use.

**Method:**

We performed a retrospective study of patients diagnosed with ASB from 2016 to 2019 at a tertiary hospital in Saudi Arabia. This study included hospitalized patients ≥ 18 years old who had a positive urine culture with no documented signs or symptoms of urinary tract infection We excluded pregnant women, solid organ transplant patients, patient on active chemotherapy, and patients about to undergo urological surgery.

**Results:**

A total of 716 patients with a positive urine culture were screened. Among these, we identified 109 patients with ASB who were included in our study. The rate of inappropriate antibiotic use was 95% during the study period. The implementation of the ASP Program was associated with a significant reduction in the use of carbapenems (*P* = 0.04) and an increase in the use of cephalosporins (*P* = 0.01). However, overprescribing antimicrobial agents was a concern in both eras. Approximately 90% of the microorganisms identified were gram-negative bacteria. Of those, 38.7% were multidrug-resistant strains.

**Conclusion:**

The urine culture order in ASB is considered relatively small number; however, it showed a high rate of the inappropriate use of antibiotics when there is an order of urine culture in both era. ASP ought to focus on targeting the ordering physician, promoting awareness and/or organizational interventions that appear to reduce the incidence of overtreatment.

## Introduction

Asymptomatic bacteriuria (ASB) refers to the isolation of ≥10^5 colony-forming units [CFU]/mL or ≥ 10^8 CFU/L of bacteria from an appropriately collected urine specimen in an individual with no signs or symptoms of urinary tract infection [1, 2]. ASB is common, but its exact prevalence is highly variable. Patients with chronic indwelling catheters and spinal cord injuries have a very high prevalence of bacteriuria (100 and 50%, respectively) compared with that among healthy premenopausal women (1%) [1–3]. In 2019, IDSA recommended against screening for and/or treating ASB with antibiotics unless patients are undergoing endoscopic urologic procedures associated with mucosal trauma or are pregnant [1]. The inappropriate treatment of ASB has no positive impact on clinical outcomes and results in adverse events [1, 4, 5]. In recent years, the overprescribing of antibiotics for ASB has contributed to an increasing number of health care-related problems in clinical practice. A prospective study found that overtreatment of ASB was responsible for 17% of prescribed antimicrobials [6]. In fact, overtreatment of ASB led to several adverse effects, such as increased prevalence of multidrug-resistant (MDR) organisms, increased rates of *Clostridium difficile* infection (CDI) and long hospitalizations, all of which factors increase the costs of health care [2, 7–9]. In addition, previous studies have shown that MDR organisms are frequently found in ASB patients, with a prevalence of 16% [4, 5, 7]. These findings support the guideline recommendations against ASB treatment. ASB is a major concern worldwide, and few studies outline the types of antibiotics unnecessarily prescribed and the associated costs. The goal of our study was to evaluate the inappropriate use of antibiotics in ASB patients before and after the implementation of Antimicrobial Stewardship Program (ASP).

## Methods

### Study setting

We conducted a 4-year retrospective, descriptive study to determine the inappropriate use of antimicrobials in ASB patients at a tertiary hospital, 700-bed located in Riyadh, Saudi Arabia.

### Antimicrobial stewardship program implementation

In 2018, ASP has been applied as one of the tools to fight microbial resistance and antimicrobial misuse. The aim was to focus on strategies that have most impact on decreasing antibiotics use and resistance. The ASP activities are perform by the stewardship team during the working hours (not weekend and after working hours). The main ASP activities include the following:

- Carbapenem restriction and prior authorization: restricted to infectious disease physicians; however, it can be given up to 24 h until ID team approve the continuation.
- Prospective audit and feedback (PAAF): The therapeutic interventions are not specific to ASB, but ASB cases were included and subjected to ASP. ASB cases interventions are through direct verbal communication with the prescriber and/or written communication in “Antimicrobial Stewardship Note) progress note.
- Education: annual infections control and antimicrobial stewardship symposium to educate clinician about appropriate use of antibiotics (ASB topic is not included in the education). In addition, antimicrobial guide is available in the hospital intranet such as clinical practice guideline in emergency department (ED) focus on ASB management where most cases were seen in ED.
- Intravenous (IV) to oral conversion

### Data collection

The data were collected from January 2016 to December 2019. The list of patients who had positive urine culture was obtained from the hospital information systemdatabase. The data collected included age, gender, drug name, dose, frequency, duration, date of treatment start and laboratory results. The criteria for selecting subjects were as follows: hospitalized patients age ≥ 18 years, with a positive urine culture and no documented signs or symptoms of urinary tract infection. The exclusion criteria included pregnancy, solid organ transplantation, active chemotherapy, and urological surgery.

MDR is defined as microorganisms, predominantly bacteria, that are resistant to one or more classes of antimicrobial agents.

### Study outcomes

This project sought to i) Evaluate the inappropriate use of antibiotics in ASB patients before and after ASP implementation; ii) assess the gaps to reduce the incidence of overtreatment; and iii) identify the type of unnecessarily prescribed antibiotics.

### Statistical analysis

Descriptive included means and standard deviations or percentages asappropriate. Post ASP impleimtation was compared to the standard of care using independent sample t-test for continuous variables and Fisher’s exact test for categorical variables.

## Result

**Fig. 1:**
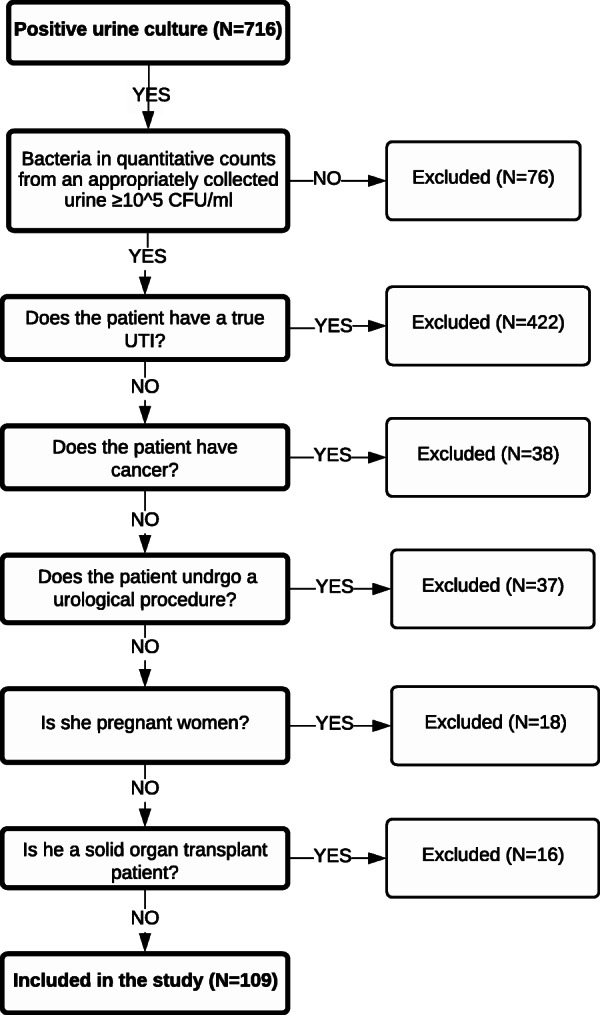
Flowchart of patient selection and inclusion and exclusion criteria

A total of 716 patients with a positive urine culture were screened. Among these, we identified 109 patients (15%) with ASB who were included in our study (Fig. 1). The median patient age was 65 years, and common comorbidities included hypertension (82%), diabetes mellitus melitis (80%), and dyslipidemia (38%) (Table 1).

**Table 1:** Show characteristics of patients diagnosed with asymptomatic bacteriuria (ASB)

Patient characteristics	Total (***N*** = 109)
Median age, in years, (range)	65 (11–98)
Female gender, n (%)	74 (68)
Urinary catheter, n (%)	64 (58.7)
**Comorbidities**	
Hypertension, n (%)	82 (75.2)
Diabetes mellitus, n (%)	80 (73.3)
Dyslipidemia, n (%)	38 (34.8)
Ischemic Heart Disease, n (%)	37 (33.9)
Neurological disease^b^, n (%)	32 (29.3)
Cerebrovascular Accident, n (%)	32 (29.3)
Chronic kidney disease, n (%)	25 (23)
Endocrine disease^c^, n (%)	13 (11.9)
Pulmonary disease^d^, n (%)	7 (6.4)
***Urinalysis result***	
Pyuria, n (%)	96 (88.0)
RBCs^e^ in urine, n (%)	36 (33.02)
Leukocytes in urine, n (%)	100 (91.74)

^a^*ASB* Asymptomatic Bacteriuria

^b^Neurological disease: Alzheimer’s, Parkinson’s, Dementia and Epilepsy

^c^Endocrine disease: Hypothyroidism and Addison’s disease

^d^Pulmonary disease: Asthma and Chronic Obstructive Pulmonary Disease

^e^*RBCs* Red Blood Cell

The most important clinically relevant finding was that 95% (*n* = 104) of ASB patients were inappropriately treated with antibiotics during the study period. Before ASP implementation, the inappropriate use of antibiotic was 96.6% (53/55) and after ASP was 94.4% (51/54). Consumption of antibiotic for ASB was 5, 3, 4 and 4 prescriptions per patient-days during the these 4 years. Approximately 71 patients (68%) were given more than one unnecessary antibiotic either concomitantly or subsequently. Also, 59% of antibiotics were administered IV, 30.4% were administered orally (PO) and 10.6% were converted from IV to PO.

The most commonly used class of antibiotics was cephalosporins (41.2%), followed by carbapenems (29%) (Table 2). Of the microorganisms identified in the urine, 90.20% were gram-negative bacteria, of those, 38.7% were MDR. The numbers of MDR cases from 2016 to 2018 and 2018–2019 were 27 (44%) and 18 (32%), respectively (Table 3).

**Table 2:** Evaluation of antimicrobial agents used, and microorganisms identified in ASB (*N* = 104)

Number of antimicrobial agents, n	151
Duration, mean	12 d
**Route of administration, n (%)**	
IV^a^	89 (59)
PO^b^	46 (30.4)
IV to PO	16 (10.6)
**Type of antimicrobial agents, n (%)**	
*Quinolones*	29 (19.2)
*β-lactam*	73 (48.3)
*Penicillin*	11 (7.28)
*Cephalosporin*	
*2nd generation*	33 (21.85)
*3rd generation*	29 (19.21)
*Carbapenem*	44 (29.14)
*Vancomycin*	1 (0.66)
*Others*	4 (2.65)
**Microorganisms identified (*N*** **= 116)**	
*Gram-positive, n (%)*	*11 (9.40)*
*Enterococcus*	7 (6.03)
*Coagulase negative staph*	2 (1.72)
*Streptococcus group B*	2 (1.72)
*Gram-negative, n (%)*^c^	*105 (90.2)*
*E. coli*	54 (46.55)
*K. pneumonie*	23 (19.83)
*Pseudomonas*	13 (11.21)
*Citrobacter*	3 (2.59)
*others*	12 (10.35)

^a^IV = intravenous; ^b^PO = orally

^c^ Multidrug Resistance was 45 (38.7%) isolates in the urine

**Table 3:** Before and after antimicrobial stewardship program implementation

	Before ASPs (***N*** = 53)	After ASPs (***N*** = 51)	***P*** Value
Number of antimicrobial agents per patient	1.3 (73/53)	1.5 (78/51)	NA
MDR, n (%)	27/61 (44%)	18/55 (32.7%)	0.20
**Antimicrobial agents, n (%)**			
Carbapenem	27/73 (40%)	17/78 (21.8%)	0.04
Cephalosporin	25/61 (37%)	37/78 (47.4%)	0.09
Quinolones	15/61 (20.5%)	14/78 (18%)	0.68

*ASPs* Antimicrobial Stewardship

*MDR* Multi-drug Resistance

## Discussion

The mismanagement of ASB is a worldwide problem. Treatment of ASB is not only useless but also harmful [1, 3–5, 10, 11]. We found that a small number of urine culture order was requested when the patient do not have documented signs and symptoms. This shows that the level of awareness in a physician is high and this practice complies with the stewardship. Implementation of ED antibiotic guideline might have an impact on this result where we found the most ASB cases in ED. Nevertheless, if the culture was requested, almost 95% of ASB cases were inappropriately treated with antibiotics, a finding that justifies this level of concern, and this percentage remained alarmingly high even after the implementation of ASP. The consumption levels were quite stable at a rate of approximately 4 prescriptions per 1000 patients-days during these 4 years. PAAF was part of the stewardship program; however, the therapeutic intervention was performed during working hours including intervention related to ASB cases. This highlights the importance of 24 h ASP coverage. Another alarming result is that the average duration of antimicrobial agents was 12 days. This duration highlights an opportunity for education, not only to avoid antibiotics for ASB, but for true UTIs, cognizance of appropriate duration of therapy.

We found a relatively high proportion of ASB among patients who were female, of advanced age, diabetic, and who had hypertension [12]. This is consistent with findings from other studies [2, 4, 7, 10].

A systematic review and meta-analysis concluded that female gender and the overinterpretation of some laboratory data (positive nitrites, pyuria, presence of gram-negative bacteria and cultures with higher microbial counts) are associated with inappropriate prescribing practices [4, 6]. In our analysis, we found a similar result.

*E. coli*, *Enterococcus* species, and *Candida* species are common bacterial and fungal colonizers of the urinary tract [13, 14]. In our analysis, *E. coli* was the most common pathogen associated with ASB, followed by *K. pneumoniae* and *Pseudomonas.* MDR bacteria are common in our patients (44% vs. 33% before and after ASP), a finding that could have an impact on the prescribing of antimicrobial agents for ASB.

Cephalosporins was the most frequently used antibiotic class (41%), followed by carbapenems (29%) and quinolones. The inappropriate carbapenem prescribing practices might be affected by the high frequency of urine MDR isolates.

The implementation of the ASP was associated with a significant reduction in the use of carbapenems (*P* = 0.04) and an increase in the use of cephalosporins (*P* = 0.1). However, overprescribing antimicrobial agents was a concern in both eras. Since the number of urine culture orders for ASB is considered relatively small number, we aim to identify whom it was ordered by (eg., intern, resident) and when it was ordered (e.g., after working hours) then target those in the stewardship. Furthermore, we plan to embed ASB educational lecture in our annual ASP conference to promote awareness among our healthcare providers and create an ASB protocol. Interestingly, the challenge of managing ASB appropriately may be surmountable with approaches such as identifying the ordering physician, promoting awareness and/or organizational interventions that appear to reduce the incidence of overtreatment.

The current study has some limitations. First, it was conducted in a single center with convenience sampling which may limit the generalizability of the findings.

## Conclusion

The rate of inappropriate use of antibiotics in ASB is high in ordered urine culture. Targeting asymptomatic bacteruria management in ASP might decrease the misuse of antibiotic, and we should focus on advocating awareness among healthcare providers and implementing stricter protocols.

## Data Availability

The data relating to this study are available from the corresponding author upon reasonable request.

## Abbreviations

CFU: Colony-forming units
IDSA: Infectious Diseases Society of America
ASB: Asymptomatic Bacteruria
ASP: Antimicrobial Stewardship Program
MDR: Multidrug-resistant
